# Editorial: Application of Cytometry in Primary Immunodeficiencies

**DOI:** 10.3389/fimmu.2020.00463

**Published:** 2020-03-19

**Authors:** Tomas Kalina, Roshini S. Abraham, Marta Rizzi, Mirjam van der Burg

**Affiliations:** ^1^Department of Pediatric Hematology and Oncology, Second Faculty of Medicine, Charles University, Prague, Czechia; ^2^Department of Pathology and Laboratory Medicine, Nationwide Children's Hospital, Columbus, OH, United States; ^3^Department of Rheumatology and Clinical Immunology, University Hospital Freiburg, Freiburg, Germany; ^4^Center for Chronic Immunodeficiency, Medical Center, University of Freiburg, Freiburg, Germany; ^5^Department of Pediatrics, Leiden University Medical Center, Leiden, Netherlands

**Keywords:** flow cytometry, primary immunodeficency, immunophenotyping, diagnosis, functional studies

Since the first commercial flow cytometers became available in the 1970s, the field of flow cytometry (flow) has seen prodigious growth. Several scientific discoveries contributed to the development of this field, including the staining properties of fluorescein by Paul Ehrlich and a systematic sizing of microscopic particles by Coulter. In the late 1960s Bonner, Sweet, Hulett and Herzenberg at Stanford University designed and patented the first Fluorescence Activated Cell Sorter (FACS) instrument, and at the same time a German researcher, Wolfgang Göhde developed a fluorescence-based flow cytometer. Since then, flow has become an essential tool not only in research but also in diagnostic settings, where it can deliver results within a few hours. Hematopathologists routinely use flow to precisely define and classify hematological malignancies, as well as to evaluate and monitor outcomes of treatment, and detect minimal residual disease (MRD) after chemotherapy or immune reconstitution after hematopoietic cell transplantation (HCT). The ability to identify, quantitate and characterize immune cells has been a continuous source of new information on development, function, and alterations in the context of disease. This is particularly true in the field of inborn errors of immunity with 416 genetic defects described, 64 of which have been identified in the past 2 years (1). In these diseases, patients present with complex changes in their immune cells (both phenotypic and functional) and the versatile nature of multiparametric flow has allowed a thorough characterization. The flow-based immunological tests range from the relatively basic and easy to adopt (lymphocyte subset quantitation –T,B, and NK cells), to the more challenging, yet standardized tests with up to 10 markers, or exploratory panels with up to 23 fluorescent markers. Another common use of flow is the detection of specific protein (surface or intracellular) whose absence leads to immunodeficiency. Additionally, flow can be used as a read-out for functional responses to a variety of signals permitting single cell evaluation. This Research Topic on Flow Cytometry for Primary Immunodeficiencies covers the spectrum of flow assays—immunophenotyping, protein detection and functional analysis, for diagnosis and monitoring of patients with these complex diseases.

While the first clinical report of a primary immunodeficiency, Bruton's agammaglobulinemia (now referred to as X-linked agammaglobulinemia) was described by Bruton (2), the discovery of a distinct subset of immune cells producing immunoglobulin was not made until 1965 by Cooper et al. (3). While some cases of agammaglobulinemia may be associated with lack of B cells, there are other cases where B cells are present but functionally defective. Flow can be used to identify patients with low or absent B cells in blood [see example of EuroFlow Primary Immunodeficiency Orientation Tube (PIDOT) performance on 94 PID cases including BTK deficiencies (van der Burg et al.)], and it can also be used to classify the specific defect based on the relative amount of individual B cell precursor populations in the bone marrow (Wentink et al.). Standardization of cytometry (4) allows for inter-laboratory comparisons, as well as for evaluation of patients over prolonged periods of time and lends itself to algorithmic evaluation. For most PIDs, a broader immunological analysis is required than mere analysis of the primary affected cell type. In Common Variable Immunodeficiency (CVID) it is well-known that other lymphocyte subsets besides B cells can be affected (5, 6). In a detailed study on predominantly antibody deficiencies (PAD), it was demonstrated that CVID patients with non-infectious complications have a mild combined immunodeficiency with defects in naïve B and T cells numbers, especially Treg, Th17, and Tfh17 subsets (Edwards et al.). Flow cytometric analysis of peripheral blood mononuclear cells from CVID patients could be extended to other cell types, which have immunomodulatory effects. Polito et al. have shown that expression of adiponectin receptors in CVID differ from controls, and changes after immunoglobulin infusion. This opens possibilities for further research on the role of adiponectin as link between the immune system and adipose tissue, which transects the boundaries of the immune system.

Diagnostic evaluation of PIDs has also been influenced by technical developments in sequencing analysis, which has resulted in much earlier introduction of genetic testing in the diagnostic process, including whole exome sequencing (WES), targeted NGS panels and whole genome sequencing, with or without a filter for PID genes (7–9). However, flow remains a crucial first step in the diagnostic process to define the immunophenotype of the patient prior to genetic analysis. In addition, flow plays an important role in interpretation or confirmation of genetic results, especially when no definitive genetic diagnosis can be made, and for functional corroboration of the phenotype. For example, a case of monozygotic twins with CVID is presented in this Research Topic with strikingly similar cytometric findings in lymphocyte subsets, yet no clear genetic variant was identified by WES (Silva et al.). Similarly, a case of loss of surface CD4 expression was described and characterized by the Euro Flow PID cytometry panels (Fernandes et al.). A clear contribution of flow to the field of PIDs is effectively reviewed by Ma and Tangye, summarizing what we have learned from phenotyping of peripheral blood lymphocytes, but also documenting the relevance of functional studies in patients with STAT3 loss-of-function (Hyper IgE syndrome) and DOCK8 deficiency. In the last decade, our conceptual understanding of primary immunodeficiencies was broadened by the realization that loss of components, protein or function is only a partial manifestation of the broader spectrum of inborn errors in immunity, and that immune dysregulation with autoimmunity and susceptibility to malignancy is the “other side of the coin”. Cabral-Marques et al. have insightfully summarized PIDs with immune dysregulation (PIRDs), their frequent immunophenotypic hallmarks, and the assays to investigate functional abnormalities. Hemophagocytic Lymphohistiocytosis, as a PIRD example has been reviewed in depth by Chiang et al.. In addition to genetic defects of the immune system, there are phenocopies of disease that are usually caused by autoantibodies to immunologically relevant molecules. Merkel et al., have summarized the functional analysis of anti-cytokine autoantibodies using flow, which is a category included in the IUIS classification of inborn errors of immunity in the last two iterations (1, 10).

Importantly, flow is indispensable in PID diagnostics in countries with limited resources. Two reviews reflect the diagnostic role of cytometry in two tertiary care centers in India (Madkaikar et al.; Rawat et al.). These articles demonstrate that while the Next-Generation Sequencing (NGS) has changed the diagnostic landscape of PIDs, and there are certain settings where WES might provide faster diagnostic discovery, there are other settings where directly evaluating presence or absence of a protein is far more rapid and economical, even though the proportion of tests with a positive PID diagnosis will be relatively small. While, those tests, which evaluate for presence or absence of specific protein (e.g., perforin, CD27, HLA DR, SAP, XIAP, LRBA, and so on) offer a fast and definitive test answer, they require a laboratory capable of performing complex flow assays with appropriate analytical and clinical validation to meet regulatory requirements. It is the experience of Indian PID centers that a validated 6-color panel can serve as a facile screen for patients with a clinical picture suggestive of a primary immunodeficiency (Rawat et al. Madkaikar et al.) and while a lack of B cells may suggest agammaglobulinemia, a lack of T cells and/or NK cells may suggest a combined immunodeficiency, or expansion CD4/CD8 double-negative T cells (DNT), a possible autoimmune lymphoproliferative syndrome (ALPS). In specialized centers, larger and standardized panels for screening and classification can facilitate rapid diagnostic decisions and guide personalized therapy. To disseminate these tests and make them more accessible, the EuroFlow consortium has built and validated several standardized operating protocols (SOPs) (11), antibody panels and they document their performance here (van Dongen et al.; van der Burg et al.).

The introduction of functional tests using flow has provided a powerful tool to dissect these genetic disorders of immunity. Immune function assays can be broadly classified into cellular proliferation, cell cytotoxicity, cellular interactions and signaling, production of biologically active molecules (cytokines, chemokines) in response to relevant stimuli, cell migration and chemotaxis as well as enzymatic assays. As an example, loss or gain of function mutations in STAT1 and STAT3 lead to different clinical phenotypes (12). The study of STAT1 phosphorylation can distinguish patients with chronic mucocutaneous candidiasis, who have gain-of-function (GOF) variants from those with Mendelian susceptibility to mycobacterial disease (MSMD) who have loss-of-function (LOF) variants in STAT1, as well as patients with pathogenic variants in the IL-12/IFN-gamma axis genes, which leads to aberrant STAT1 phosphorylation. STAT1 phosphorylation by flow can not only be used diagnostically but also to monitor the response to targeted therapy, making precision medicine for PIDs a reality (13). In addition, flow cytometric evaluation of STAT1 phosphorylation was shown to be of benefit in a diagnostic algorithm for patients with *Talaromyces marneffei* infection in HIV negative children (Lee et al.). In this Research Topic, van Zelm et al., demonstrate a STAT3-dependent activating defect in B-cells of a patient with a STAT1 GOF mutation, once again proving the power of single cell analysis. While numerous assays, which measure T-cell proliferation are available, Bitar et al., have developed a rapid *in vitro* assay evaluating STAT-5 phosphorylation as a proxy for T-cell proliferation in immunodeficiency. Also, a new actin polymerization assay was developed by Kopitar et al., which established the pathogenicity of a variant in ARPC1B deficiency, and could potentially be used in other diseases affecting actin filament formation (e.g., Wiskott-Aldrich syndrome).

While the regulations overseeing diagnostic flow varies depending on the country and the healthcare system, the burden of ensuring a high standard of data is universal (14, 15). Flow assays pose unique validation challenges due to the nature of the measurements being made and the measurands—cellular analysis in a variety of biological matrices, variability in quantifying and interpreting results are some of the confounders (16). Recent efforts to standardize research and clinical flow have come in the form of “minimum information” guidelines—MIFlowCyt (The Minimum Information about a Flow Cytometry Experiment) (17), MiSet RFC standards (Minimum Set of Research Flow Cytometry Standards) and OMIPs (Optimized Multicolor Immunofluorescence Panel) (18). Therefore, each laboratory has to determine their repertoire of testing, the type and volume of patient samples and ability to effectively develop and validate various flow immunophenotyping and functional assays (19, 20).

In the field of clinical and human immunology, where availability of biological material is limited, especially from pediatric patients, flow offers significant and granular detail on cell composition, cellular diversity, and functional response at the cellular subset level from a relatively small sample volume. This is particularly relevant in the field of rare diseases where identification of single gene disorders must be complemented by a proof of altered immune function and phenotype, both quantitatively and qualitatively (21–23).

As much as acquisition of immunological data from biological samples by flow requires consideration of multiple aspects, data analysis represents another significant frontier to be tackled. This is most relevant for multiparametric flow data, where complex immunophenotyping panels are used to interrogate large patient cohorts. Ellyard et al. have used heatmaps to analyze 54 parameters (cell subsets) from a cohort of 276 individuals who were normalized to controls using centile distribution. This approach allows for rapid identification of cell subsets, which are either abnormally expanded or contracted in a particular patient group. Similarly, the EuroFlow PIDOT tube was applied to 99 genetically defined PID cases and a heatmap of cellular subsets was produced to visualize abnormal patterns. Attardi et al. used a series of Principal Component Analysis (PCA) plots to delineate and visualize anomalies found in a cohort of 100 PID patients, while Emmaneel et al., used a cohort of 78 antibody-deficient cases compared to 100 controls to build a pipeline for computational identification of cases and controls using FlowSOM clusters, random forest analysis or support vector machines. Thus, automated data analysis and exploration of large cohorts for PID-related features are becoming a rich resource enhancing the understanding of complex immunological characteristics.

In the future, we are likely to see further advances in flow, in technology and instrumentation, but also in throughput and data analysis. Techniques that are currently confined to the research realm, such as mass cytometry or spectral cytometry, are likely to find their way to the diagnostic laboratory, with applications in practical clinical immunology. Further, there will also be improvements in reagents, specifically for human immunology, allowing higher degrees of standardization within and between laboratories. The next decade promises to be exciting and innovative for flow, especially for immune-mediated diseases.

## Author Contributions

All authors listed have made a substantial, direct and intellectual contribution to the work, and approved it for publication.

### Conflict of Interest

The authors declare that the research was conducted in the absence of any commercial or financial relationships that could be construed as a potential conflict of interest.
